# *Staphylococcus lugdunensis*: antimicrobial susceptibility and optimal treatment options

**DOI:** 10.1007/s10096-019-03571-6

**Published:** 2019-05-29

**Authors:** Lana Taha, Marc Stegger, Bo Söderquist

**Affiliations:** 1 School of Medical Sciences, Faculty of Medicine and Health, Örebro University, Örebro, Sweden; 2 Department of Bacteria, Parasites and Fungi, Statens Serum Institut, Copenhagen, Denmark; 3 Department of Laboratory Medicine, Clinical Microbiology, Örebro University Hospital, SE-701 85 Örebro, Sweden

**Keywords:** Staphylococcus lugdunensis, Antibiotic susceptibility testing, Penicillin G, Oxacillin

## Abstract

*Staphylococcus lugdunensis* is a coagulase-negative staphylococcus (CoNS) with unusual pathogenicity resembling that of *S. aureus.* Unlike other CoNS, *S. lugdunensis* remains susceptible to most antibiotics. The resistance to penicillin varies widely (range, 15–87% worldwide), whereas methicillin resistance is still rare. We aimed to evaluate treatment options for infections caused by *S. lugdunensis* and more specifically to investigate whether penicillin G could be a better treatment choice than oxacillin. Susceptibility testing was performed using the disc diffusion method for penicillin G, cefoxitin, trimethoprim/sulfamethoxazole, erythromycin, clindamycin, gentamicin, norfloxacin, fusidic acid, rifampicin, and fosfomycin. Isolates susceptible to penicillin G were further tested with a gradient test for penicillin G and oxacillin. Of the 540 clinical isolates tested, 74.6% were susceptible to penicillin G. Among these penicillin-susceptible isolates, the MIC_50_ and MIC_90_ values for penicillin G were threefold lower than that for oxacillin. A majority of the isolates were susceptible to all other antibiotics tested. Breakpoints for fosfomycin have not yet been defined, and so no conclusions could be drawn. Two isolates were resistant to cefoxitin and carried the *mecA* gene; whole-genome sequencing revealed that both harbored the SCC*mec* element type IVa(2B). *S. lugdunensis* isolated in Sweden were susceptible to most tested antibiotics. Penicillin G may be a more optimal treatment choice than oxacillin. Although carriage of the *mecA* gene is rare among *S. lugdunensis*, it does occur.

**Electronic supplementary material:**

The online version of this article (10.1007/s10096-019-03571-6) contains supplementary material, which is available to authorized users.

## Introduction

*Staphylococcus lugdunensis* is a coagulase-negative staphylococcus (CoNS) with a significant pathogenic potential compared with other CoNS. First described in 1988 [1], it is part of the normal skin flora and is most commonly found in the groin area, lower extremities, and axilla [2, 3], that is, in areas possessing excess apocrine sweat glands [4]. However, it can also be frequently found in the nasal cavity [5]. Although a commensal, *S. lugdunensis* has the ability to cause aggressive infections, resembling those of *S. aureus* rather than of other CoNS. *S. lugdunensis* is a common cause of skin and soft tissue infections [4, 6, 7], but can also cause bone and joint infections [8], native and prosthetic valve endocarditis [9, 10], and bacteremia [11, 12].

*S. lugdunensis* remains remarkably susceptible to most antibiotics, unlike many other CoNS such as *S. epidermidis.* Resistance to penicillin has been reported to be as low as 15.5% and 20% in Sweden and Denmark, respectively [4, 13], but considerably higher in the USA (45%) [14] and Taiwan (87%) [15]. Resistance to erythromycin and clindamycin is overall very low [4, 14]; Taiwan is the exception, with reported resistance levels of 17–25% [15, 16]. According to a review by Argemi et al. [17], susceptibility to fosfomycin is greatly variable, reporting resistance in > 50% of isolates. However, breakpoints for fosfomycin have not yet been defined. The prevalence of methicillin resistance and presence of the *mecA* gene ranges from 0 to 8.3% [14, 18–21], with the exception again being Taiwan, where the reported occurrence is 21% [16]. For detection of *mecA*, cefoxitin disc breakpoints are a better option than oxacillin disc breakpoints, which have lower sensitivity and specificity [22]. The breakpoints for cefoxitin and oxacillin are the same as for *S. aureus* (www.eucast.org).

Until recently, the methods used to distinguish different CoNS species from *S. aureus* were based on phenotypical properties, such as the DNase and coagulase tests [9]. Moreover, the differentiation between *S. lugdunensis* and other staphylococci has been based on the presence of ornithine decarboxylase activity (ODC) and a pyrrolidonyl arylamidase test [14, 22] that shows positivity in more than 90% of *S. lugdunensis* isolates [23]. However, although *S. lugdunensis* does not produce secreted coagulase, it can sometimes be mistaken for *S. aureus* at clinical microbiology laboratories. This is explained by the fact that up to 65% [24] of *S. lugdunensis* isolates possess a membrane-bound form of coagulase (clumping factor) that shows a positive result in the latex agglutination test and slide coagulase test [1, 9, 23]. Since the implementation of MALDI-TOF MS, the identification of *S. lugdunensis* has become much easier, more accurate, and more cost effective for routine laboratories [25].

The first line of antimicrobial treatment of staphylococcal skin and soft tissue infections is isoxazolyl penicillin [26, 27], especially in areas with a low prevalence of MRSA. In Sweden, the prevalence of MRSA in blood cultures displaying positivity for *S. aureus* is still approximately 1%. In addition, if a majority of *S. lugdunensis* also displays full susceptibility to penicillin, it would be of interest to evaluate whether penicillin could be a better treatment option than oxacillin for those penicillin-susceptible isolates, in order to optimize the treatment for infections caused by *S. lugdunensis* and thereby reduce selection of isoxazolyl penicillin–resistant staphylococci. We therefore aimed to compare the MIC values for penicillin and isoxazolyl penicillin in penicillin-susceptible isolates of *S. lugdunensis* and to determine the antibiotic susceptibility pattern of *S. lugdunensis* for multiple antimicrobial agents.

## Materials and methods

### Bacterial isolates

From January 2010 to October 2014 and from November 2017 to January 2018, all isolates that were identified as *S. lugdunensis* from clinical samples at the Department of Laboratory Medicine, Clinical Microbiology, Örebro University Hospital, Sweden, were saved. MALDI-TOF MS (Microflex LT and Biotyper 3.1, Bruker Daltonik, Bremen, Germany) was implemented at this department in January 2014, which made it possible to perform an accurate identification of *S. lugdunensis*; before this, staphylococcal species were identified by screening with DNase and coagulase tests. The presence of ODC was tested in order to distinguish *S. lugdunensis* from other CoNS, and since some isolates of *S. epidermidis* also show positivity for ODC, further testing was performed with trehalose-mannitol broth to distinguish *S. lugdunensis* from *S. epidermidis.* Final species determination was in some cases performed by using API ID32 Staph (bioMérieux, Marcy l’Etoile, France).

All isolates were stored at − 80 °C in preservation medium (Trypticase Soy Broth, BD Diagnostic Systems, Sparks, MD, USA, supplemented with 0.3% yeast extract, BD Diagnostic Systems, and 29% horse serum, SVA, Uppsala, Sweden) and subcultured overnight at 36 °C on Mueller-Hinton agar (BD Diagnostic Systems). Isolates that did not have the phenotypical appearance, color, or typical smell of *S. lugdunensis* were retested with MALDI-TOF MS.

### Susceptibility testing

Susceptibility testing for ten antibiotics was performed with the disc diffusion method according to the 2017 EUCAST guidelines [28]. The cultures were suspended in sterile saline to 0.5 McFarland and inoculated on Mueller-Hinton agar plates (BD Diagnostic Systems). A maximum of three antibiotic discs per agar plate were put in place and inoculated for 18 h ± 2 h at 35 °C. Ten antibiotics were tested (all discs from Oxoid, Basingstoke, UK): penicillin G (1 IU), cefoxitin (30 μg), clindamycin (2 μg), norfloxacin (10 μg), trimethoprim-sulfamethoxazole (25 μg), erythromycin (15 μg), gentamicin (10 μg), fusidic acid (10 μg), rifampicin (5 μg), and fosfomycin (50 μg). Cefoxitin (30 μg) discs were used as a screening test for oxacillin resistance.

All isolates susceptible to penicillin G were further tested with a gradient test (Etest, bioMérieux) for penicillin G and oxacillin, also according to EUCAST guidelines. Identification of the *mecA* gene was performed on those isolates resistant to cefoxitin, using Genie II and eazyplex MRSAplus (Amplex Diagnostics GmbH, München, Germany).

### WGS and analysis of SCC*mec*

Genomic DNA was extracted from the *mecA*-positive isolates using the DNeasy Blood and Tissue Kit (Qiagen, Hilden, Germany), with subsequent library construction using the Nextera XT Kit (Illumina, Little Chesterford, UK) before whole-genome sequencing was performed using a 300-cycle kit on the NextSeq platform (Illumina) according to the manufacturer’s instructions. Resistance genes were identified with ARIBA [29], using raw reads against the ResFinder database (https://cge.cbs.dtu.dk/services/ResFinder/). The raw reads were assembled using version 3.10.1 of SPAdes [30] and used for MLST typing with the MLST command-line tool (https://github.com/tseemann/mlst). SCC*mec* types were identified using version 1.2 of SCC*mec*Finder [31].

## Results

**Fig. 1:**
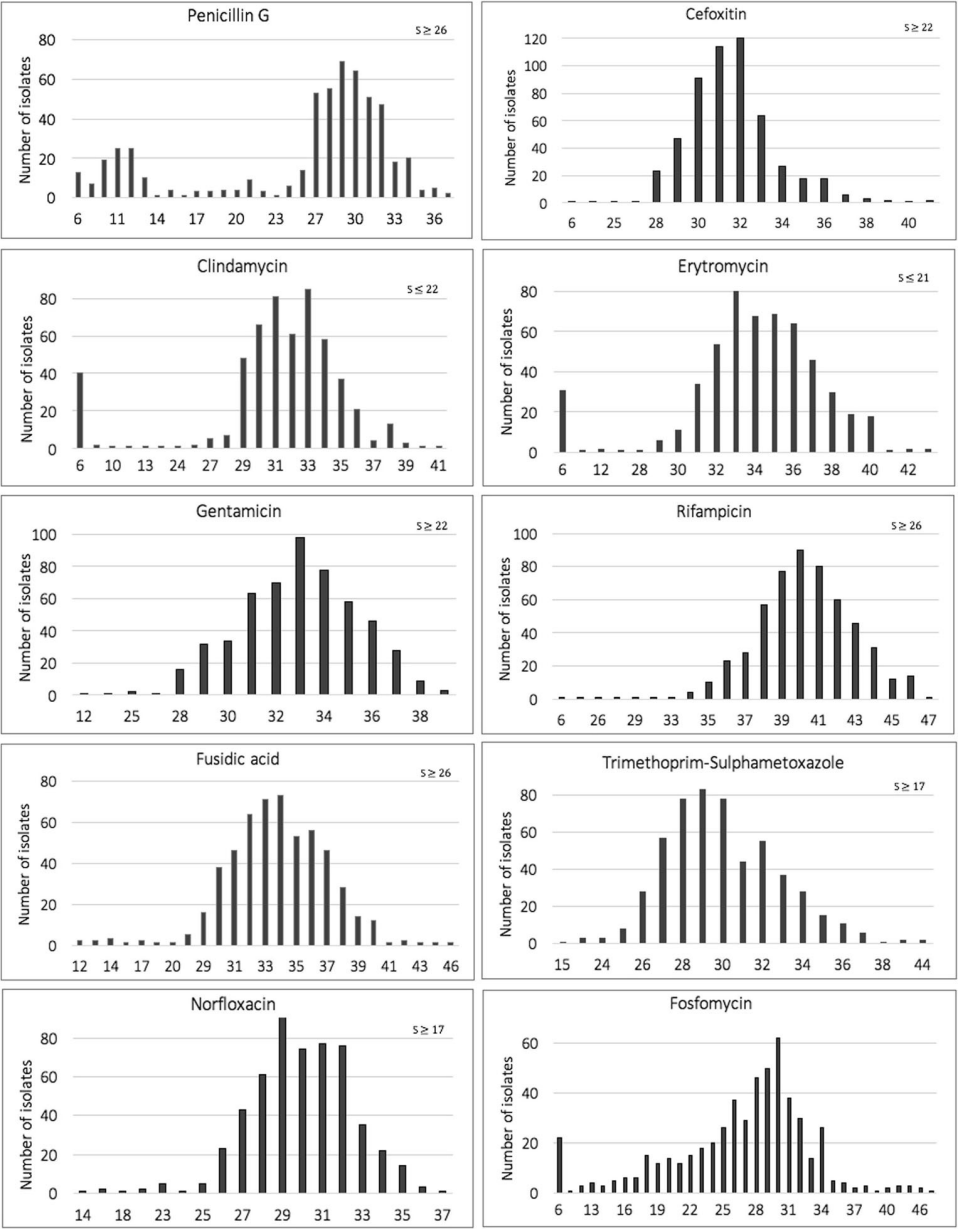
Zone diameters in millimeters (x-axis) for all antimicrobial agents tested against 540 *Staphylococcus lugdunensis* isolates using the disc diffusion method

The samples included in the present study were mainly from primary and secondary infections of skin and soft tissue structures and consisted of 569 isolates. Of these, 29 isolates were either non-viable or had been misidentified as *S. lugdunensis*. The site of sampling, etiology of the misidentified isolates, and number of isolates included from each year are given in Supplementary files (Tables S1A–S3A). Antibiotic susceptibility testing on the remaining 540 isolates revealed that 25.4% (137/540) were resistant to penicillin G (Table 1). Furthermore, there was also a trend towards decreasing resistance during the time period (chi-squared test for trend; *p* < 0.001; Table S3A). Two isolates that were resistant to cefoxitin (oxacillin MIC > 256 mg/L and 32 mg/L respectively) and carried the *mecA* gene were whole-genome sequenced in order to characterize the SCC*mec* (see below). One isolate was resistant to norfloxacin but remained susceptible to all other antibiotics. Of the two isolates that were resistant to rifampicin, one was also resistant to trimethoprim-sulfamethoxazole; and of the two isolates resistant to gentamicin, one was also resistant to fusidic acid. A total of 34 isolates were resistant to erythromycin, and all but one also showed resistance to clindamycin. Zone diameters for all antibiotics tested by the disc diffusion method are presented in Fig. 1. Although as previously mentioned there are as yet no defined breakpoints for fosfomycin, the median of the zone diameter was 28 mm and 85.7% of the isolates had a zone diameter larger than 20 mm (Fig. 1).

**Table 1:** Antibiotic susceptibility pattern of 540 Staphylococcus lugdunensis isolates tested with the disc diffusion method

Antibiotic	No. (%) susceptible
Penicillin G	403 (74.6)
Gentamicin	538 (99.6)
Rifampicin	538 (99.6)
Cefoxitin	538 (99.6)
Fusidic acid	528 (97.8)
Trimethoprim/sulfamethoxazole	539 (99.8)
Norfloxacin	539 (99.8)
Clindamycin	494 (91.5)
Erythromycin	506 (93.7)

**Fig. 2:**
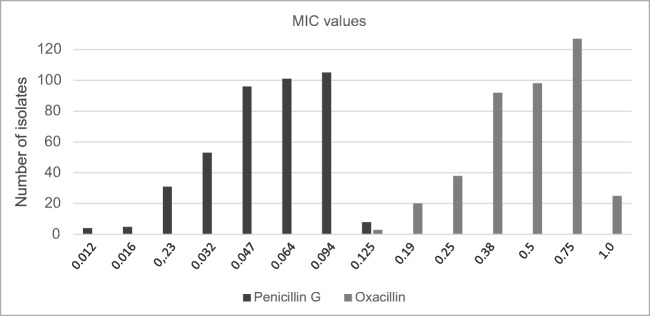
Minimum inhibitory concentration (mg/L) values for penicillin G and oxacillin in the 403 penicillin G–susceptible isolates among all 540 *Staphylococcus lugdunensis* isolates tested with the disc diffusion method. Etest was used for gradient testing.

The MIC values determined by a gradient test for penicillin G were all lower than (or the same as) those for oxacillin (Fig. 2). MIC_50_ was 0.064 mg/L for penicillin G and 0.5 mg/L for oxacillin, whereas MIC_90_ was 0.094 mg/L for penicillin G and 0.75 mg/L for oxacillin. In general, the MIC for penicillin G was three dilution steps lower than that for oxacillin (*p* < 0.0001 paired *t* test). Whole-genome sequencing of the two *mecA*-positive *S. lugdunensis* to an average depth of 100 or above revealed that they were ST38 and ST44 according to the MLST typing scheme. Both isolates harbored the SCC*mec* type IVa(2B), but with variations within the J3 region. Further analysis of the resistome identified *erm*(C), *tet*(K), and *blaZ* in the ST38 isolate, whereas the ST44 only carried *blaZ* in addition to *mecA*. These findings were in concordance with the phenotypic results.

## Discussion

As a coagulase-negative staphylococcus, *S. lugdunensis* remains remarkably susceptible to various antimicrobial agents. In the present study, conducted in Örebro, Sweden, the clinical *S. lugdunensis* isolates were almost fully susceptible to all tested antibiotics. However, resistance levels reported from other parts of the world indicate the emergence of resistance. A Taiwanese study reported resistances to penicillin and oxacillin of 87% and 20%, respectively [15], while another study from the USA found that 45% of *S. lugdunensis* isolates were resistant to penicillin [14]. By comparison, our results showed a resistance level of 25.4% regarding penicillin G, indicating that the isolates in our study remain mainly naïve or of wild type. Furthermore, there was rather a trend towards decreasing resistance levels throughout the sample period. However, this species has the potential to become increasingly resistant, predominantly by acquiring the *blaZ* gene encoding penicillinase production, but other similar resistance mechanisms could be acquired. In addition, a case report by Kragsbjerg et al. [32] described the development of resistance to rifampicin and ciprofloxacin during treatment of a persistent *S. lugdunensis* infection in a patient with septic arthritis, infective endocarditis, and vertebral osteomyelitis.

Despite the fact that *S. lugdunensis* is susceptible to oxacillin in vitro, treatment failure is not uncommon [33], indicating that oxacillin may be suboptimal in some cases. As previous studies have shown, the prevalence of *mecA*, and thereby methicillin resistance, is not yet extensive among *S. lugdunensis* [14, 17–21]. The identification of SCC*mec* in two highly distant ST types provides evidence of multiple acquisitions, and whereas the whole-genome sequencing indicated overall the same type of SCC element (type IVa(2B)), they were of different origin based on their J3 differences. Regarding the other resistance genes in the two multidrug- and methicillin-resistant *S. lugdunensis* isolates, analyses revealed a chromosomal integration of *blaZ* based on coverage, whereas the *tet*(K) and *erm*(C) were both carried on a small 4.5 kb plasmid previously found in both *Staphylococcus aureus* strain USA300 FPR3757 [34] and *Staphylococcus epidermidis* strain ATTC 12228 [35].

As noted in the present study, penicillin-susceptible *S. lugdunensis* displayed lower MIC values for penicillin G than for oxacillin, with a difference of approximately three dilution steps between their MIC_50_ as well as the highest MIC values. From a clinical point of view, this implies that a lower concentration (i.e., lower dosage) of penicillin G is needed for a therapeutic effect in comparison with oxacillin. Unnecessarily high doses of antibiotics may not only increase the risk of side-effects in individual patients but could also have adverse effects on the macro- and microenvironment such as the skin and the gut microbiome. Differences in pharmacokinetics offer another reason why penicillin G could be a better choice of treatment. Isoxazolyl penicillin has a higher serum protein binding capacity (94–98%) than penicillin G (65%) [36], which means that higher doses of the drug are needed to reach therapeutic levels in order to achieve optimal concentrations of a free fraction of the antimicrobial agent at the infection site since antibacterial efficacy is correlated to T > MIC.

Regarding fosfomycin, resistance among *S. lugdunensis* has only been described in a single paper [17]. Furthermore, there are no current international or national breakpoints for fosfomycin. Our results did not show a distinct difference between a supposed wild population and isolates displaying decreased susceptibility or resistance. When tested for fosfomycin, the two sequenced isolates displayed zone diameters of 20 mm and 26 mm respectively, and neither of them harbored *fosA/fosB* genes.

One limitation of the present study is that no data were available regarding the clinical course of disease or the severity of the infections. This limitation could be addressed in a prospective randomized trial comparing the efficacy of penicillin G/V versus cloxacillin/flucloxacillin.

The method used in this study is based on a standardized protocol and was performed according to the EUCAST guidelines. All laboratory work and the determinations were performed by one individual, eliminating the potential bias of performing and interpreting the susceptibility tests and gradient tests differently, and thus reducing inter-person variability. The major strength of this study lies in the number of isolates investigated, predominantly from skin and soft tissue infections, and the inclusion of present-day isolates.

Although knowledge has increased dramatically in the past decade, investigations into the diversity, virulence, and population structure of *S. lugdunensis* are still in their infancy. Like other CoNS, *S. lugdunensis* has the ability to produce biofilm in various degrees, and despite being highly susceptibility to most antimicrobial agents, once biofilm is produced an infection becomes significantly more difficult to treat [18]. However, despite the presence of the *icaADBC* locus, which encodes polysaccharides central in biofilm production in most staphylococcal species, it is not used in the formation of biofilm by *S. lugdunensis.* Instead, the biofilm displayed by this species is more proteinaceous [37]. The autolysin/adhesion AtlL plays a role in biofilm formation of *S. lugdunensis* but also acts as a virulence factor by binding to extracellular matrix proteins and by promoting internalization [38]. In addition, the surface protein sortase A seems to be important in the pathogenesis of infective endocarditis [39]. Another recent discovery by Zipperer et al. [40] is that some isolates of *S. lugdunensis* have the ability to produce lugdunin, a novel antibiotic compound that inhibits the growth of *S. aureus,* other Gram-positive bacteria, and even vancomycin-resistant enterococci*.* This finding could offer the possibility of development of new antimicrobial agents.

Our results indicate that penicillin is a better option than oxacillin for treating *S. lugdunensis* infections. A review of the literature reveals that a great majority of *S. lugdunensis* still remain susceptible to penicillin in the Western world. This is beneficial since penicillin G is a narrow spectrum antibiotic which has advantageous pharmacokinetics as well as MIC values that are three-fold lower than oxacillin, as shown in the present study. This finding should advocate the inclusion of penicillin G as a routine for antibiotic susceptibility testing of suspected CoNS or verified *S. lugdunensis* by clinical microbiological laboratories.

## Conclusion

In the present study, performed in Sweden, the majority of *S. lugdunensis* isolates were susceptible to all tested antibiotics. Almost 75% of the isolates were susceptible to penicillin G. Among these, the MIC values for penicillin G were threefold lower than that for oxacillin, indicating that penicillin G may be a better treatment choice; a conclusion which may also be supported by pharmacokinetic data. Carriage of the *mecA* gene among *S. lugdunensis* was rare.

## Electronic supplementary material

ESM 1 (DOCX 37 kb)
