# Supplementary material for: Staphylococcus lugdunensis: antimicrobial susceptibility and optimal treatment options
Source: Eur J Clin Microbiol Infect Dis. 2019 May 29;38(8):1449–55. doi: 10.1007/s10096-019-03571-6 (PMC6647525; doi:10.1007/s10096-019-03571-6)
Supplement: Supplementary file 1 — (DOCX 37 kb) [file 10096_2019_3571_MOESM1_ESM.docx]

**Supplementary files**

| **Table S1A.** *Site of sampling for 540* Staphylococcus lugdunensis *isolates that underwent susceptibility testing in Örebro, Sweden (2010–2014 and 2017–2018).* | |
| --- | --- |
| Sample sites | No. of isolates |
| Primary skin and soft tissue infections | 19 |
| Post-operative infections | 51 |
| Wound infections | 204 |
| Deep tissue infections | 25 |
| Abscesses | 39 |
| Urine | 39 |
| Synovial fluids | 13 |
| Ear secretions | 18 |
| Implant/prosthetic device associated infections | 3 |
| Other body fluids | 7 |
| Unknown | 122 |

| **Table S2A.** *Aetiology of misidentified* Staphylococcus lugdunensis *isolates, re-evaluated by MALDI-TOF MS.* | |
| --- | --- |
| Species | No. of isolates |
| *S. aureus* | 15 |
| *Bacillus simplex* | 1 |
| *Kocuria* spp*.* | 1 |
| *S. hominis* | 1 |
| *S. simulans* | 4 |
| *S. warneri* | 1 |
| Non-viable isolate | 5 |
| Unknown spp. | 1 |
| Total | 29 |

| **Table S3A**. *Distribution of* Staphylococcus lugdunensis *samples over years, and numbers (%) susceptible to penicillin G.* | | |
| --- | --- | --- |
| Years | No. of isolates | No. (%) susceptible |
| 2010 | 85 | 75 (88) |
| 2011 | 99 | 77 (78) |
| 2012 | 126 | 92 (73) |
| 2013 | 101 | 78 (77) |
| 2014 | 12 | 7 (58) |
| 2017 | 88 | 53 (60) |
| 2018 | 29 | 21 (72) |
| Total | 540 | 403 (75) |
